# Development of Theranostic Cationic Liposomes Designed for Image-Guided Delivery of Nucleic Acid

**DOI:** 10.3390/pharmaceutics12090854

**Published:** 2020-09-08

**Authors:** Hai Doan Do, Christine Ménager, Aude Michel, Johanne Seguin, Tawba Korichi, Hélène Dhotel, Corinne Marie, Bich-Thuy Doan, Nathalie Mignet

**Affiliations:** 1 Université de Paris, UTCBS (Chemical and Biological Technologies for Health Unit), CNRS, INSERM, 75006 Paris, France; hai_doan.do@etu.parisdescartes.fr (H.D.D.); johanne.seguin@parisdescartes.fr (J.S.); tawba.korichi@etu.parisdescartes.fr (T.K.); helene.dhotel@parisdescartes.fr (H.D.); corinne.marie@parisdescartes.fr (C.M.); 2 Chimie ParisTech PSL Research University, Institute of Chemistry for Life and Health Sciences, i-CleHS, SEISAD, F-75005 Paris, France; bich-thuy.doan@chimieparistech.psl.eu; 3 Sorbonne Université, Laboratoire PHENIX (Physicochimie des Electrolytes et Nanosystèmes Interfaciaux), CNRS, 4 Place Jussieu, F-75005 Paris, France; christine.menager@sorbonne-universite.fr (C.M.); aude.michel@upmc.fr (A.M.); 4 Chimie ParisTech, PSL Research University, F-75005 Paris, France

**Keywords:** magnetic cationic liposome, nucleic acid delivery, magnetofection, MRI, magnetic targeting

## Abstract

Cationic liposomes have been considered as potential vectors for gene delivery thanks to their ability to transfect cells with high efficiency. Recently, the combination of diagnostic agent and therapeutic agents in the same particle to form a theranostic system has been reported. Magnetic liposomes are one of these examples. Due to the magnetic nanoparticles encapsulated in the liposomes, they can act as a drug delivery system and, at the same time, a magnetic resonance imaging contrast enhancement agent or hyperthermia. In this work, nucleic acid delivery systems based on magnetic cationic liposomes (MCLs) were developed. Two different techniques, reverse phase evaporation and cosolvent sonication, were employed for liposome preparation. Both strategies produced MCLs of less than 200 nm with highly positive charge. Enhancement of their transverse and longitudinal relaxivities *r_2_* and *r_1_* was obtained with both kinds of magnetic liposomes compared to free magnetic nanoparticles. Moreover, these MCLs showed high capacity to form complexes and transfect CT-26 cells using the antibiotic-free pFAR4-luc plasmid. The transfection enhancement with magnetofection was also carried out in CT26 cells. These results suggested that our MCLs could be a promising candidate for image-guided gene therapy.

## 1. Introduction

In the late 1980s and early 1990s, gene therapy has revolutionized in the care of rare diseases by proposing to use a gene as a medicine [1,2,3]. Nowadays, gene therapy has been proposed for many type of diseases, such as cancer [4]. Gene therapy can be simply defined as the genetic modification of cells to produce a therapeutic effect [5]. Theoretically, in gene therapy, a healthy gene is used to replace the distorted one or a new gene is introduced to express a therapeutic protein [6]. However, the large molecular weight and anionic charge of nucleic acid prevent it from reaching the site of action and providing its effect. Therefore, the therapeutic gene must be introduced into cells through a vehicle or vector. Since it was developed for the first time in 1989 by Rosenberg [2], viral vectors are still the most efficient vehicle for gene delivery thanks to their high transfection efficiency and ability to incorporate the delivered gene into the host genome [7]. Although there has been a lot of innovation to minimize their side effects, viral vectors still present some disadvantages such as difficulty of mass production, high cost, low stability and limited size of the nucleic acid that can be delivered. To overcome these drawbacks, non-viral vectors are still being studied. A non-viral vector is composed of numerous types of materials from inorganic ones, such as metal nanoparticles, carbon nanotubes, or graphene to organic ones such as liposomes, polymersomes, micelles, or dendrimers [7]. Among them, cationic liposomes are considered as potential candidates for gene delivery owing to their capacity to carry a large-size gene and be produced at a large scale at reasonable cost [8,9]. Moreover, they recently reached the clinic by carrying small interfering RNA (siRNA) in the case of Onpattro® (patisiran) for the treatment of Hereditary Transthyretin-mediated Amyloidosis (hATTR), which is a progressively incapacitating and often fatal genetic disorder.

Cationic liposomes contain positively charged lipids that are able to form complexes with the therapeutic nucleic acid through ionic interactions. Moreover, these vehicles are also able to encapsulate therapeutic/diagnostic agents in their aqueous cavity or in the lipid bilayer. In our lab, we have synthesized various cationic lipids and found that the most efficient one for gene transfection in vitro is 2-(3- [bis(3-aminopropyl)amino]propylamino)-*N*-ditetradecyl- carbamoylmethylacetamide (DMAPAP) [10,11]. The cationic liposome formulation composed of DMAPAP and 1,2-dioleoyl-sn-glycero-3-phosphoethanolamine (DOPE) (50/50 mol/mol) was shown to be as efficient as lipofectamine in vitro [12]. More advanced formulations composed of DMAPAP showed siRNA-based inhibitory expression in vivo [13,14].

Although cationic liposomes show promising results for gene transfection in vitro, their low transfection efficiency in vivo still limits their clinical application. To overcome this limitation, magnetic cationic liposomes (MCLs) could be a solution [15,16,17]. In MCLs, magnetic nanoparticles (MNPs) are encapsulated inside the water core of the liposome. MNPs is introduced in many drug delivery systems thanks to their biodegradability, safety, and ease of being functionalized [18,19]. With magnetic response, MNPs-based drug delivery systems can accumulate at the target site of action using an external magnet [20]. Moreover, MNPs are also able to enhance contrast for T2 weighted magnetic resonance imaging (MRI) and produce heat under an alternative magnetic field which can be applied for MRI contrast enhancement or hyperthermia [18,19,20].

Previously, we successfully prepared ultra-magnetic liposomes (UMLs) by a reverse phase evaporation method, which gave a higher MNPs loading efficiency (about 100 folds) compared to the classical thin film hydration method [20,21]. Thanks to high content of MNPs, these vehicles are able to give high T2 MRI contrast enhancement and targeting effect in vivo [22]. Based on these positive results, in this work, we ought to obtain magnetic lipoplexes that would benefit from targeting efficacy and MRI contrast enhancement. The first challenge was to obtain positively charged liposomes containing a high MNP content that is able to condense DNA while being colloidally stable. Reverse phase evaporation and cosolvent sonication were optimized to prepare MCLs for magnetic guided gene delivery. To study transfection and magnetofection efficiency, a pFAR4 plasmid encoding luciferase was chosen and condensed in MCLs. pFAR4 is a small gene vector that does not carry any antibiotic resistance marker. Finally, the efficacy of the MCLs as MRI contrast agents was shown.

## 2. Materials and Methods

### 2.1. Materials

**Figure 1:**
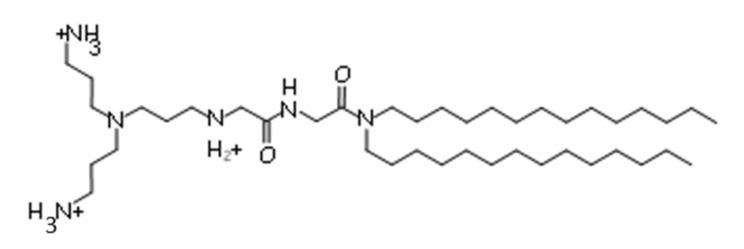
Chemical structure of 2-(3-[bis(3-aminopropyl)amino]propylamino)-*N*-ditetradecyl-carbamoylmethylacetamide (DMAPAP).

DPPC (1,2-dipalmitoyl-sn-glycero-3-phosphocholine), DSPC (1,2-distearoyl-sn-glycero-3- phosphocholine), DOPE (1,2-dioleoyl-sn-glycero-3-phosphoethanolamine), 18:0 PEG2000 PE (1,2-distearoyl-sn-glycero-3-phosphoethanolamine-*N*-[methoxy(polyethylene glycol)-2000]) and 14:0 PEG750/1000/2000 PE (1,2-dimyristoyl-sn-glycero-3-phosphoethanolamine-*N*- [methoxy(polyethylene glycol)-750/1000/2000]) were purchased from Avanti Polar lipids (Alabaster, AL, United States). Cationic lipid DMAPAP (2-(3-[bis(3-aminopropyl)amino]propylamino)-*N*-ditetradecyl -carbamoylmethylacetamide) (Figure 1) was synthesized in our lab as described in the [12] methods. The CT26 murine colon carcinoma cell line (ATCC, CRL-2638) was purchased from American Type Culture Collection (LGC Standards, Molsheim, France). CT26 cells were cultured in complete Dulbecco’s modified Eagle’s medium GlutaMAX (DMEM) (Thermo Fisher Scientific, Waltham, MA, United States) containing 10% fetal bovine serum (FBS) (Sigma-Aldrich, St. Louis, MO, United States), penicillin (50 U/mL), and streptomycin (50 U/mL) (Thermo Fisher Scientific, Waltham, MA, United States) with 5% CO_2_ at 37 °C.

Transfection efficiency was monitored using a plasmid free of antibiotic resistance marker, pFAR4-CMV-LUC (pFAR4-luc) (prepared in our group) that encodes the luciferase reporter protein expressed from the cytomegalovirus (CMV) promoter [23]. Quant-iT^TM^ PicoGreen® was purchased from Invitrogen (Carlsbad, CA, United States), the Luciferase assay system was purchased from Promega (Madison, WI, United States), the Pierce™ BCA Protein Assay Kit was from Thermo Fisher Scientific (Waltham, MA, United States), and Resazurin sodium salt was from Sigma-Aldrich (St. Louis, MO, United States).

**Figure 2:**
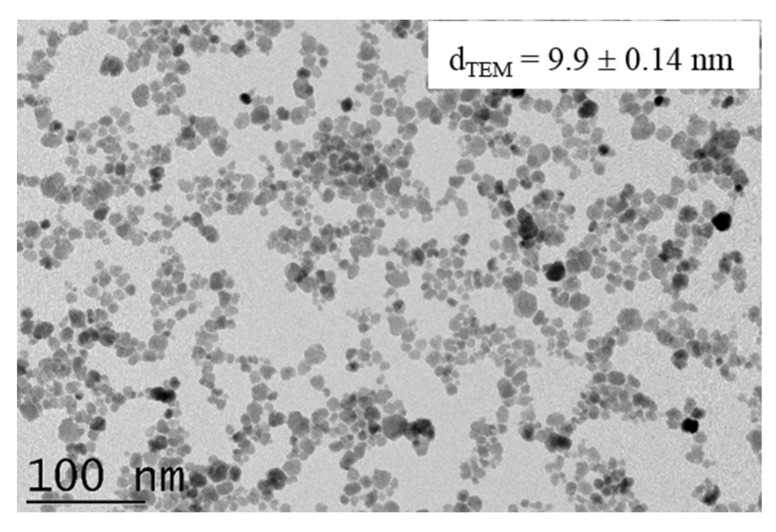
TEM image of γ-Fe_2_O_3_ magnetic nanoparticles (MNPs).

Citrate-coated γ-Fe_2_O_3_ (cit-Fe_2_O_3_) MNPs used in this work was synthesized by the co-precipitation method as described in our previous studies [22] (Figure 2, Table S1). All the lipids were dissolved in CHCl_3_ (Carlo Erba Reagents, Val-de-Reuil, France) at a concentration of 25 mg/mL.

### 2.2. Cationic Liposome Preparation

Cationic liposomes composed of DOPE, DMAPAP, and PEG-PE (49/50/1 molar ratio) were formed by the EtOH injection method as described in reference [12]. DMAPAP, DOPE, and PEG-PE, which were well dissolved in EtOH, were mixed together (total lipid = 5 µmol). The final volume of lipid solution was adjusted to 500 µL. In the meantime, 5 mL of Mili-Q water in a round-bottom flask was stirred at 700 rpm. Next, the EtOH solution of lipids was dropped on the stirring water and left to stir for 5 h. After that, EtOH was removed from the mixture by a rotary evaporation. The evaporation process was controlled to make sure that the suspension did not foam and was stopped when it reached the appropriate volume (about 1 mL). The size and zeta potential of the liposomes were measured, and the liposomes were stored at 4 °C for further experiments.

### 2.3. Magnetic Liposome Preparation

#### 2.3.1. Reverse Phase Evaporation Method

**Figure 3:**
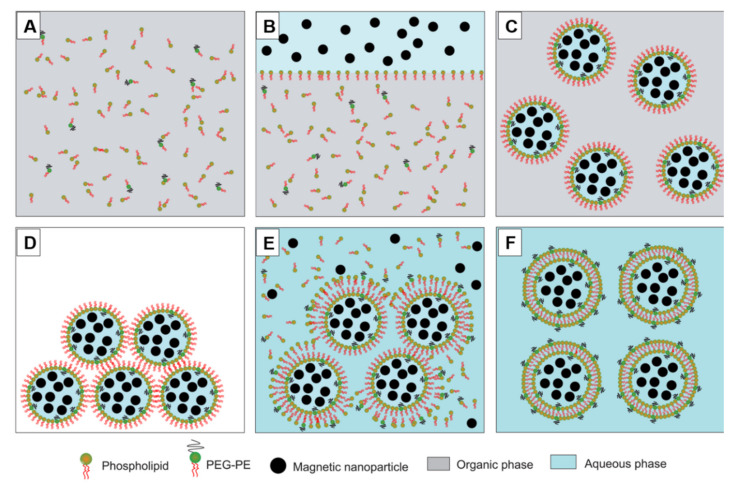
Diagram of ultra-magnetic liposomes (UMLs) or MCLs preparation by reverse phase evaporation method. Phospholipids were dissolved in organic phase (CHCl_3_ + diethyl ether) (**A**); then, an aqueous phase containing MNPs was added (**B**); After 20 minutes of sonication, a water-in-oil emulsion was formed (**C**); Then, the organic solvent was evaporated, leading to the formation of a gel form (**D**); At the critical point, some vesicles were broken, and the excess phospholipid in the environment interacted with the residual micelles to complete the lipid bilayer, (**E**) leading to the formation of liposomes (**F**).

The reverse phase evaporation method was employed to prepare UMLs and MCLs. Generally, the two preparations were based on the same protocol (Figure 3), the difference was about the lipid components and the concentration of MNPs (Table 1). All lipids were dissolved in 1 mL of CHCl_3_ and well mixed with 3 mL of diethyl ether. Then, MNPs in 1 mL of distilled water were added. Thanks to a 20 min sonication process, a water-in-oil emulsion was obtained, which was then was rapidly transferred to a rotavapor to evaporate the organic solvent at 28 °C. After all the organic solvent was removed, a gel phase was formed temporarily before the break of some droplets and formation of liposomes. Then, the mixture was kept at low pressure for 5 more minutes to remove all of the solvent. After that, big aggregates were removed by filtration through a 0.45 µm filter while free lipids and non-encapsulated MNPs were eliminated by two magnetic sorting with a strong NdFeB magnet (150 × 100 × 25 mm).

#### 2.3.2. Post-Insertion of DMAPAP into Pre-Formed UML

**Figure 4:**
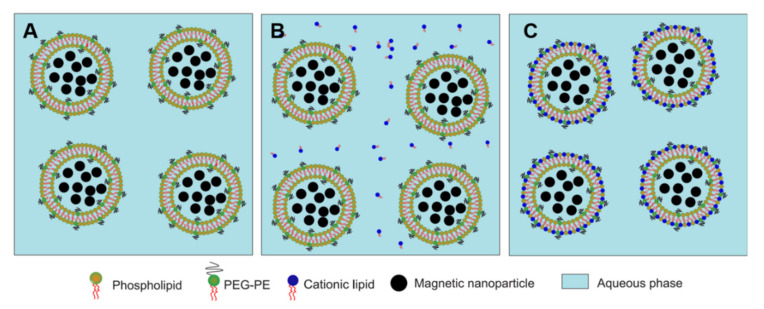
Diagram of MCLs preparation by post insertion method. Pre-formed UMLs were dispersed in H_2_O (**A**); then, a solution of DMAPAP in H_2_O was added (**B**); Hydrophobic interaction between the liposome bilayer and the acyl-chain of DMAPAP led to the insertion of DMAPAP into the phospholipid bilayer (**C**).

For post insertion of DMAPAP, a solution of DMAPAP (5 mg/mL; the volume of DMAPAP solution is dependent on the desired molar ratio of DMAPAP) in distilled water was added to diluted UMLs (2 mL, [Fe] = 50 mM, total lipid = 0.36 μmol). The mixture was stirred for 1 h. After that, large aggregates were removed by centrifugation following by a magnetic separation to get rid of free lipids (Figure 4).

#### 2.3.3. Cosolvent Sonication Method

**Figure 5:**
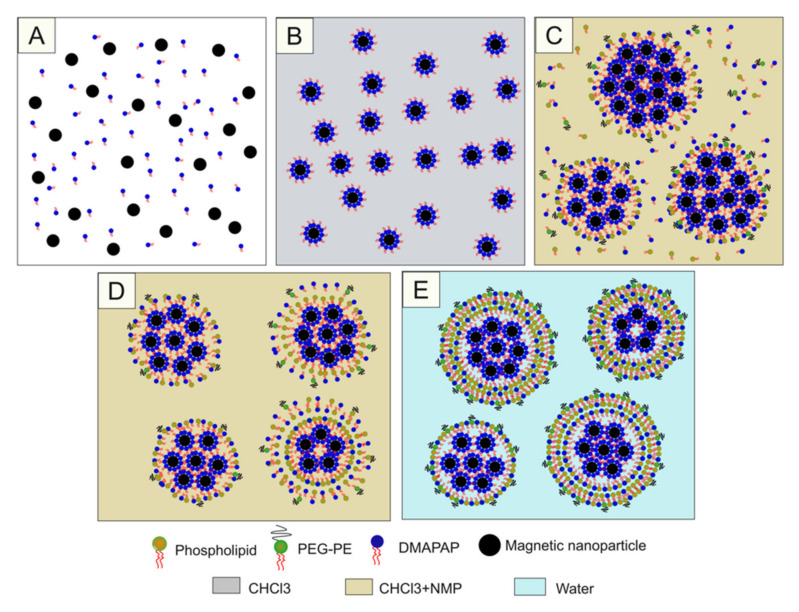
Diagram of MCLs preparation by cosolvent sonication method. Cit-MNPs and DMAPAP were mixed at an equal molar ratio in H_2_O (**A**); The aggregates (FF-DMAPAP) were collected and washed with ethanol before dispersion in CHCl_3_ (**B**); Next, FF-DMAPAP were mixed with other lipids in CHCl_3_. Then, a second solvent, *N*-methyl pyrolidone (NMP), was added (**C**); Due to a long period of sonication, a clear suspension was obtained (**D**); After the evaporation of CHCl_3_, addition of H_2_O, and dialysis, the cationic magnetic liposomes were formed (**E**).

In this approach, we formed hydrophobic MNPs composed of cit-Fe_2_O_3_ and DMAPAP (FF-DMAPAP). These hydrophobic NPs were utilized to fabricate MCLs by the cosolvent sonication method [15]. In brief, cit-MNPs and DMAPAP were mixed at an equal molar ratio in H_2_O. The aggregates (FF-DMAPAP) were collected and washed with ethanol before dispersion in CHCl_3_. Next, FF-DMAPAP (50 μL, [Fe] = 1 M) were mixed with other lipids (5 μmol in total of DOPE, DMAPAP, and PEG-PE at a molar ratio of 49/50/1) in 0.5 mL of CHCl_3_. Then, a second solvent, *N*-methyl pyrolidone (NMP) (1 mL), was added. After adding NMP, the mixture became opaque. After a long period of sonication (1 h, 3 h or 6 h), a clear suspension was obtained. Then, CHCl_3_ was evaporated, and 3 mL of distilled water was added. NMP and free lipids were removed from the MCLs by dialysis against water (MWCO 12–14 kDa) overnight at room temperature (Figure 5).

### 2.4. Lipoplex Formation

A pFAR4 plasmid encoding luciferase (pFAR4-luc) was used to study the lipoplex formation and transfection efficiency of our MCLs. Since DMAPAP has 3 positive charges, and 1 µg of DNA has 3 nmol of negatively charged phosphate, in this work, the charge ratio (RC), which is defined as the moles of positive charge per moles of negative charge (N/P) was replaced by an equivalent value of nmol of DMAPAP per µg of pFAR4-luc. Typically, lipoplexes of various charge ratios (RC 8, 10, 12) were obtained by the dropwise addition of an equal volume of diluted pFAR4-luc (in 10% Glucose, 40 mM NaCl) into a diluted solution of MCLs or positive control (cationic liposomes DOPE:DMAPAP:C14PEG1000 1%) at various cationic lipid concentrations in water. The lipoplexes were left at room temperature for 30 min before use.

### 2.5. Characterization

#### 2.5.1. Dynamic Light Scattering

The hydrodynamic diameters and zeta potential of our liposomes were determined in water (viscosity of 0.8872 cP, refractive index (*RI*) of 1.330). Meanwhile, for stability test, liposomes or lipoplexes were dispersed in complete medium (DMEM + 10% FBS + penicillin + streptomycin, viscosity of 0.9400 cP, *RI* of 1.345) and incubated at 37 °C. Dynamic light scattering (DLS) measurement was carried out at indicated time points. Dynamic Light Scattering (Nano ZS, Malvern, United Kingdom) was set up with detection angle at 173° and automatic optimization of conditions.

#### 2.5.2. Atomic Absorption Spectroscopy

The amount of iron in the MCLs was determined by flame atomic absorption spectroscopy using an AAnalyst 100 device (Perkin Elmer, Waltham, MA, United States). A calibration curve was obtained between 0.5 and 2.5 mg/L in iron in nitric acid HNO_3_ 2% by measuring the absorbance at 480 nm. Then, 5–10 μL of sample was degraded in 500 µL of 37% HCl; then, it was diluted about 500 times in HNO_3_ 2% to be in the concentration range of the calibration.

Encapsulation efficiency (*EE*) *EE* = $\frac{Fe_{2}O_{3}\left(MCLs\right)}{Fe_{2}O_{3}\left(in\right)}\times 100\%$ and loading efficiency (*LE*) *LE* = $\frac{Fe_{2}O_{3}\left(MCLs\right)}{Fe_{2}O_{3}\left(MCLs\right)+LPs}\times 100\%$ of iron oxide nanoparticles was calculated using the given formulas where *Fe_2_O_3(in_*_)_ is the initial amount of *Fe_2_O_3_* put into liposomes (g), *Fe_2_O_3(MCLs_*_)_ is the amount of Fe_2_O_3_ in the MCLs (g), and *LPs* is the amount of total lipids put into liposomes (g).

#### 2.5.3. Transmission Electron Microscopy (TEM) and Transmission Electron Cryomicroscopy (cryoTEM)

Images of MCLs were obtained with TEM. Briefly, 10 µL of the sample at 1 mM of iron was deposited on a carbon-coated copper grid. After 2 min, excess solution was removed by a filter paper, and the grid was left to be dried at room temperature. Next, the grid placed on a slide that was inserted in the microscope. The grid was analyzed with a JEOL JEM 100 S (JEOL Ltd. Tokyo, Japan) TEM operating at 80 kV. TEM images were captured using an Orius Sc 200 digital Camera (Gatan-Roper Scientific, Evry, France).

To see better the structure of the MCLs, cryoTEM was carried out. First, 4 µL of MCLs at 10 mM of iron were deposited on a carbon copper grid. Then, a guillotine system was used to rapidly absorb the droplet and plunge the grid in liquid ethane to form a 100 μm film. Imaging was performed using a JEOL 2010 microscope (JEOL Ltd. Tokyo, Japan).

#### 2.5.4. Relaxivity at 7 T

Five samples were prepared at molar concentration of iron $\left(\left[Fe\right]\right)$ = 0, 0.05, 0.1, 0.2, 0.5, and 1 mM in individual 200 μL volume of water solution in 200 μL Eppendorf tubes. The longitudinal and transverse relaxivities $r_{1}$ and $r_{2}$ of the magnetic particles dispersed in water were measured using $T_{1}$ and $T_{2}$ maps at 7 T, on a 300 WB Bruker microimaging spectrometer. The acquisition parameters are as follows: for *T_1_* map: RARE images; *TE* = 13 ms; *TR* = 15 s, 8 s, 3 s, 1.2 s, 0.8 s, 0.594 s, 0.3 s, 0.144 s, 0.05 s, 0.033 s, RARE factor 2; for *T_2_* map: multi-echo MSME images: hermitian pulse, *TR/TE* = 15 s/11 ms, 32 echos. Fields of view of 3 × 3 cm^2^, a matrix size of 128 × 64, and a slice with a thickness of 1.5 mm were used for *T*_1_ and *T*_2_ maps. Relaxation times *T_1_, T_2_,* and *T_2_* * of each sample were calculated by fitting:

$$\mathrm{for}\;T_{1}:\;y=A+C\;\times \;(1-\exp\left(\frac{-t}{T_{1}}\right))$$

$$\mathrm{for}\;T_{2}\;\mathrm{and}\;T_{2}*:\;y=A+C\;\times \;\exp\left(\frac{-t}{T_{2}}\right)$$

In this range, the inverse of the relaxation times was found to vary linearly with $\left[Fe\right]$. Molar relaxivities *r_1_* and *r_2_* in mM^−1^.s^−1^ were computed using the following equation:

$$\frac{1}{T_{y}}=r_{y}\left[Fe\right]+\frac{1}{T_{y,0}}$$

with *y*: 1, 2, or 2 *; $T_{y,0}$ denotes the longitudinal and transverse relaxation time of water.

#### 2.5.5. Lipoplex Formation Efficiency

The lipoplexation efficiency of the MCLs was evaluated by PicoGreen^®^ assay. The lipoplexes were prepared as described above. PicoGreen is a fluorescent probe that binds double stranded DNA (dsDNA) and forms a highly luminescent complex compared to the free dye in solution. To perform this assay, the supernatants of lipoplexes after magnetic sedimentation were diluted in TE buffer (200 mM Tris-HCl, 20 mM EDTA, pH 7.5); then, 50 µL of this diluted solution was mixed with 50 µL of Quant-iT^TM^ PicoGreen^®^ dsDNA reagent solution (diluted 200 times in TE buffer) in a black 96-well plate, followed by 4 min incubation in dark. Free pFAR4-luc plasmid with exact concentration in the range of 0.027–27 ng/mL was used to carry out a calibration curve. The fluorescence of the samples was read with a microplate reader (TECAN Infinite F200 Pro, Männedorf, Switzerland) (excitation at 485 nm and emission at 535 nm). The amount of free pFAR4-luc plasmid in the lipoplexes was calculated using a calibration curve. Lipoplexation efficiency was calculated using the following equation:

$$\mathrm{Lipoplexation}\;\mathrm{efficiency}\;\%\;=\;\frac{\left(DNAi-DNAf\right)}{DNAi}\times 100\%$$

where *DNA_i_* is the initial amount of pFAR4-luc plasmid put into the lipoplexes and *DNAf* is the amount of free pFAR4-luc plasmid.

### 2.6. Transfection In Vitro

CT26 colon carcinoma cells were cultured in complete DMEM containing 10% FBS, penicillin (50 U/mL), and streptomycin (50 U/mL) with 5% CO_2_ at 37 °C. Cells were seeded on a 96-well plate at density of 10^4^ cells/100 µl/well 24 h before the transfection. On the day of transfection, 100 µL of either fresh medium (for control) or lipoplex (1 µg pFAR4-luc/well) was loaded on each well. A 0.28 T magnet (Chemicell, Berlin, Germany) was placed under the plate in case of magnetofection. The medium was changed after the indicated time, and the transfection was completed after 24 h.

To evaluate the transfection efficiency, luciferase and protein assays were performed. Cells were washed twice with PBS and then lysed with a Luciferase Cell Culture Lysis Reagent (Promega, Madison, WI, United States). Next, a white plate containing 10 µL of cell lysate supernatant/well was placed into the Tecan luminometer. The luminometer was set up to automatically inject 50 µL of Luciferase Assay reagent per well and the light produced was measured as relative light unit (RLU). Besides, the protein content in cell lysate was also quantified using a Pierce™ BCA Protein Assay Kit (Thermo Fisher Scientific, Waltham, MA, United States). Then, 10 µL of cell lysate supernatant or albumin standard solution in lysis buffer was treated with 10 µL of iodoacetamide 0.1 M at 37 °C for 1 h. Then, the mixture was left to react with bicinchoninic acid (BCA) working reagent for about 45 min at 37 °C before revealing the absorbance at 562 nm. The luciferase level was expressed as RLU/µg of protein.

### 2.7. Cytotoxicity Test

Cytotoxicity tests were performed on a CT26 cell line and TIB75 cell line using Alamar blue assay. This assay is based on a reaction of living cells to reduce non-fluorescent resazurin in fluorescent red resorufin.

Briefly, cells were plated in a 96-well plate at a concentration of 2 × 10^4^ cells/100 µL/well 24 h before the test. Then, the medium was replaced by 100 µL of lipoplex/MCLs or free pFAR4-luc at various concentration of iron or pFAR4-luc. The cells were continued to be incubated at 37 °C/5% CO_2_ for another 24 h. Next, 20 µL of resazurin 0.15 mg/mL was added to each well. After 4 h of incubation, the fluorescence (λex = 530 nm; λem = 590 nm) was measured in each well with a microplate reader (TECAN Infinite F200 Pro, Männedorf, Switzerland). The viability was calculated as following:

$$\mathrm{Viability}\%\;=\;\frac{F_{sample}-mean\left(F_{blank}\right)}{mean\left(F_{solvent}\right)-mean\left(F_{blank}\right)}\times \;100\%$$

where *F_sample_, F_blank_* and *F_solvent_* represent the fluorescence of the well treated with the formulation, well without cells, and well with cells treated with the solvent of lipoplex (5% Glucose, NaCl 20 mM), respectively.

### 2.8. Statistical Analysis

Graph Pad software (version 8.0.2, 2019, San Diego, California, United States) was used to analyze the data and determine the statistical significance between groups. Data are shown as mean ± standard deviation (SD). A one-way analysis of variance (ANOVA) test was used for multiple comparisons and a *p* value ≤ 0.05 was considered as significant. The symbol meaning: ns non-significant *p* > 0.05, * *p* ≤ 0.05, ** *p* ≤ 0.01, *** *p* ≤ 0.001, **** *p* ≤ 0.0001.

## 3. Results and Discussion

### 3.1. MCLs by Post Insertion of DMAPAP

**Figure 6:**
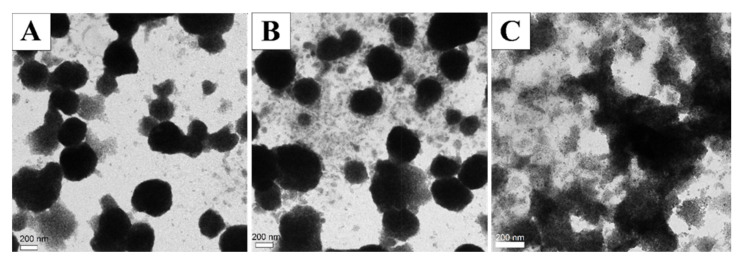
TEM images of ultra-magnetic liposomes (UMLs): (**A**) before and (**B**) after insertion of 10% or (**C**) 40% of 2-(3-[bis(3-aminopropyl)amino]propylamino)-*N*-ditetradecyl- carbamoylmethylacetamide (DMAPAP). 10% or 40% of DMAPAP per total lipid was added to a diluted dispersion of UMLs (50 mM of Fe, 0.36 μmol total lipids). After stirring for 1 h, centrifugation, and magnetic separation, TEM images of the post-inserted UMLs was observed.

As we had previously optimized the preparation of high MNPs-loaded magnetic liposomes, our first idea to obtain positively charged magnetic liposomes was to post-insert cationic lipids to preformed magnetic liposomes. Post insertion (or post modification) of liposomes is a known technique to insert polyethylene glycol (PEG) lipid into the outer layer of pre-formed liposomes [24]. This process is spontaneous, and it is driven mainly by the hydrophobic interaction of the membrane components and the PEG lipid. Therefore, the advantage of this technique is that the lipid inserts at the surface of the liposomes do not affect the encapsulated components. First, we added 10% of DMAPAP (per total lipids) to the preformed liposomes and observed that after the insertion of DMAPAP, the liposome retained the same structure (Figure 6A,B). The zeta potential was slightly increased from −45 to −38 mV, which is not sufficient for DNA interaction (Table S2). Increasing the amount of DMAPAP to 20% and 40% DMAPAP/total lipids dramatically impacted the structure of liposomes and did not allow obtaining positively charged magnetic liposomes, independently of the medium or the pH used (Figure 6C, Figures S1 and S2, Tables S2 and S3).

It was reported in other work that to achieve a high modification ratio, the post-insertion method should be performed above the phase transition temperature [25]. As the phase transition temperature of our UMLs (DPPC/DSPC/18:0 PEG2000 86/9/5 molar ratio) is 43 °C [26], the post-insertion of 40% DMAPAP (per total lipids) was performed at 45 °C and at room temperature. Similar results were obtained; no temperature effect was evidenced (Table S4).

These results suggested that the post-insertion method could only be applied to add a small amount of DMAPAP into preformed liposomes (10% of total lipids). The bilayer of liposomes did not resist to the addition of a higher amount DMAPAP. As our objective was to obtain positively charged MCLs, this method was not considered as a good strategy.

### 3.2. Optimization of MCLs by Reverse Phase Evaporation Method

In order to form MCLs, we turned to the initial protocol based on reverse phase evaporation, which allowed us to obtain magnetic liposomes with 1 M iron. However, due to the strong interaction between cationic lipids and anionic MNPs, liposomes would not form, even at various pH (data not shown). These results pointed out that a high concentration of MNPs was not suitable for MCLs preparation, mainly leading to large aggregates. As a consequence, the ratio between Fe and DMAPAP had to be optimized to obtain stable cationic liposomes. Various formulations of MCLs were prepared by increasing the iron concentration from 2.5 to 25 mM, meaning that the molar ratio of Fe/DMAPAP varied from 2 to 20.

**Figure 7:**
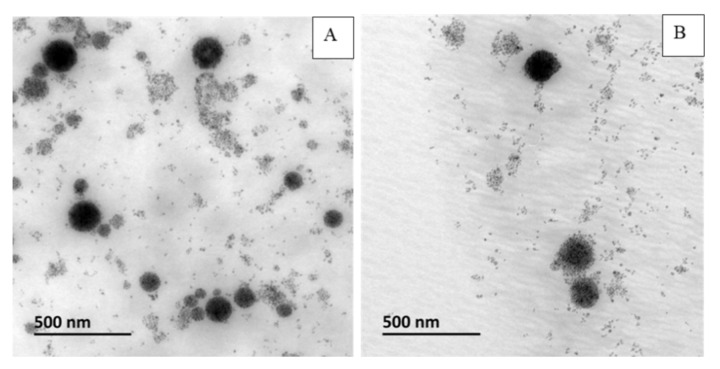
TEM image of MCLs prepared with: (**A**) 2.5 mM (REV_MCL_2.5) or (**B**) 10 mM (REV_MCL_10) of iron.

From the results in Table 2, we see that when only 2.5 mM of Fe was used, very small and homogenous liposomes of about 130 nm were formed with high positive charge. When we increased the amount of Fe, the size of the liposome increased, while the zeta potential decreased. In TEM images, magnetic liposomes full of encapsulated MNPs were observed (Figure 7). With 25 mM of Fe, big and inhomogeneous liposomes of nearly 400 nm were obtained. When a higher concentration of MNPs was used (50 mM), only aggregates with negative charges were formed [data not shown].

The hydrodynamic size and zeta potential of MCLs were measured 3 times/sample, the mean and SD of three means of the 3 measurements were given. The encapsulation and loading efficacy of MNPs in the liposomes were calculated as described above. The data given are the averages of 3 different experiments (*n* = 3); bars, SD.

When the iron concentration was less than or equal to 10 mM, a similar encapsulation efficiency of more than 70% was observed, leading to a proportional increase of the loading efficiency to the input Fe concentration. A high amount of MNPs of 25 mM caused some aggregates, which were removed during the filtration, resulting in a low encapsulation efficiency (35%).

Based on these results and the fact that a high amount of MNPs loaded in the liposomes will be necessary for MRI imaging and magnetic targeting, we selected the formulation with the initial concentration of 10 mM in iron for further studies. The formulation will be named REV_MCLs.

### 3.3. Optimization of MCLs by Cosolvent Sonication Method

During the experiment, we found that cationic lipid DMAPAP can interact with anionic cit-Fe_2_O_3_ MNPs to form aggregates. Taking advantage of this interaction, we formed hydrophobic MNPs composed of cit-Fe_2_O_3_ and DMAPAP (FF-DMAPAP). These hydrophobic NPs were utilized to fabricate MCLs by the cosolvent sonication method. The second solvent NMP was used as it is miscible with both CHCl_3_ and water. NMP can also help LPs to stabilize on the surface of FF-DMAPAP NPs [15].

#### 3.3.1. Effect of Sonication Time

**Figure 8:**
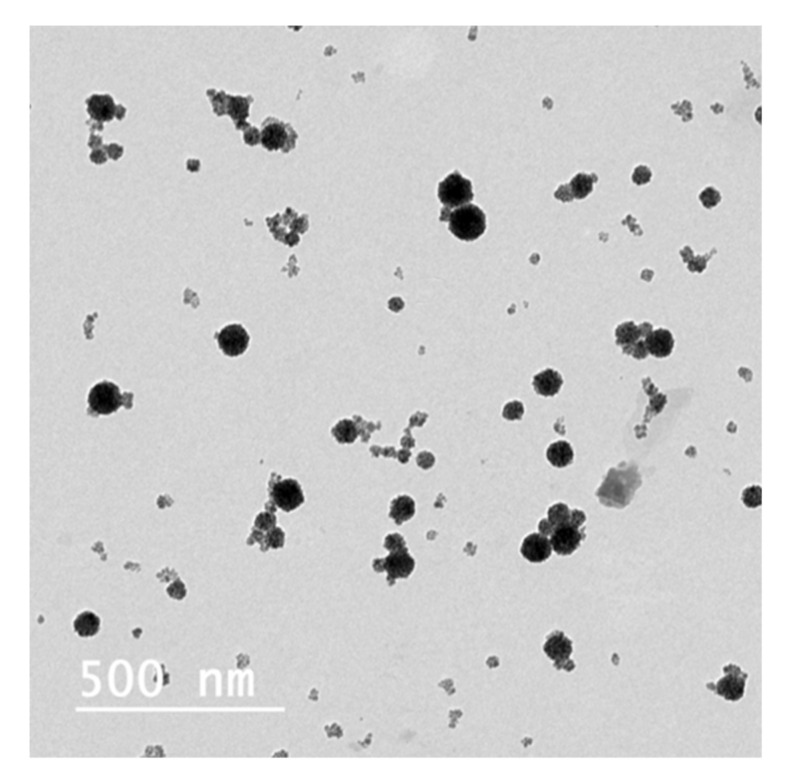
TEM images of cosol_MCL_3 h.

This method was developed from the study of Jiang et al., in which they used lipid-like molecules (lipidoid) to coat the surface of hydrophobic MNPs. They found that one of the most important factors that can affect the quality and transfection efficiency of lipidoid-coated MNPs was the sonication time. After 6 h of sonication, they obtained clusters of about 300 nm. A longer sonication time led to much smaller particle size down to about 50 nm after 8 h [15]. To understand the role of sonication time in MCLs formation and characterization, in our study, three different samples with various sonication times of 1 h, 3 h, and 6 h were prepared. From Table 3, we found that the longer the sonication time was, the smaller the size and PDI of the liposome was. Small liposomes of about 200 nm were obtained after 3 h of sonication (Figure 8). When we continued sonicating for 3 more hours, the liposome size decreased, but not much. So, we chose a sonication time of 3 h for further studies.

Hydrodynamic size and zeta potential were measured 3 times per sample; the mean and SD of three means of the 3 measurements were given. The encapsulation and loading efficacy of iron oxide nanoparticles in liposomes were calculated as described above. The data given are the averages of 3 different experiments (*n* = 3); bars, SD.

#### 3.3.2. Effect of PEG_n_-PE on the Colloidal Stability of Cosol Formulations

PEGylation has been well known for its ability to obtain a ‘stealth’ effect. It was proven that PEG inhibits protein binding on the liposome surface, leading to the reduction of opsonization and therefore prolonging its circulation time. Moreover, the influence of PEGylation on the half-life time of liposomes depends on both the PEG chain length and their density on the surface [24]. The stealth effect of PEG for cationic liposomes has been questioned in some publications, as there were some evidences that PEGylation did not prolong the half-life time of cationic nanoparticles [27]. Moreover, the presence of PEG on the liposome surface can inhibit the interaction between the particles and cell membrane, leading to less cellular uptake and/or release [24,28]. However, to maintain the colloidal stability of our magnetic cationic liposomes, PEG was needed. To avoid these drawbacks, we chose to incorporate a small amount of PEG with low PEG length. In order to evaluate the PEG length required to maintain the stability of MCLS, various PEGs with the same lipid chain were tested: 14:0 PEG750 PE, 14:0 PEG1000 PE and 14:0 PEG2000 PE.

**Figure 9:**
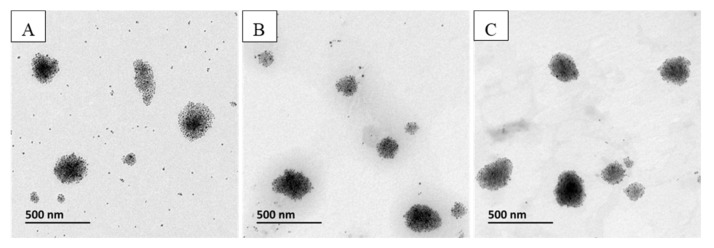
TEM images of MCLs prepared by the cosolvent sonication method with various PEG-PE: (**A**) 14:0 PEG750 PE; (**B**) 14:0 PEG1000 PE; (**C**) 14:0 PEG2000 PE.

As we can see in Table 4, the presence of PEG-PE on the MCLs surface influences the liposome size as the MCL hydrodynamic size increased when the PEG length increased. However, the size of MCLs observed by TEM was independent on the length of PEG (Figure 9). This discrepancy might be explained by the fact that the hydrodynamic size of liposomes is measured by DLS, while non-hydrated liposomes are observed by TEM. The hydration layer around the liposomes is strongly influenced by the insertion of PEG chains, which interact strongly with the water molecules in the medium.

The data given are the averages of 2 different experiments (*n* = 2). Hydrodynamic size and zeta potential were measured 3 times/sample, the mean and SD of the means of the 3 measurements were given; bars, SD.

**Figure 10:**
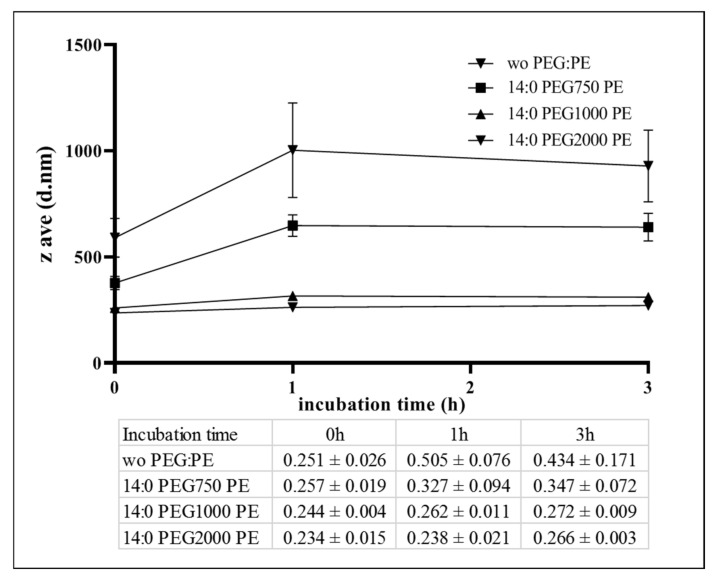
Dynamic size and PDI of cosol_MCLs in cell culture medium after incubation at 37 °C. Cosol_MCLs with various PEG-PE (14:0 PEG750-PE, 14:0 PEG1000-PE, 14:0 PEG2000-PE) or without PEG-PE (wo PEG-PE) were diluted 5 times in complete Dulbecco’s modified Eagle’s medium (DMEM) containing 10% fetal bovine serum (FBS), penicillin (50 U/mL), and streptomycin (50 U/mL), and incubated at 37 °C. Hydrodynamic size was measured by dynamic light scattering after 0 h, 1 h or 3 h incubation. The inserted table presents PDI value of the samples. The data given are averages of 3 different experiments (*n* = 3); bars, SD.

In addition, the PEG length did not really influence the MCL zeta potential but strongly affected the MCL stability in cell culture medium (Figure 10). As shown, without the presence of PEG-PE in the formulation, the magnetic liposomes formed aggregates of about 600 nm, since they were mixed with the culture medium containing FBS. After 1–3 h of incubation, the aggregates became bigger and reached nearly 1 µm. Meanwhile, the PEG750-PE was able to prevent protein binding but not completely. It can be observed from Figure 10 that the aggregation happened right after the incubation of MCLs in FBS containing DMEM, leading to the appearance of large particles of about 400 nm. These aggregates grew up to about 630–650 nm after incubation at 37 °C for 1–3 h. However, the size of these aggregates was always smaller than that without PEG:PE. In contrast, PEG1000-PE and PEG2000-PE stabilized the MCLs by preventing protein binding. The MCL size was measured at around 250–300 nm and remained stable 3 h post-incubation at 37 °C in the presence of FBS. Therefore, we pursued our study with MCLs containing 1% of PEG1000-PE in further studies. The sample will be named cosol_MCLs.

### 3.4. Comparison of MCLs Prepared by Two Methods

#### 3.4.1. TEM, CryoTEM

**Figure 11:**
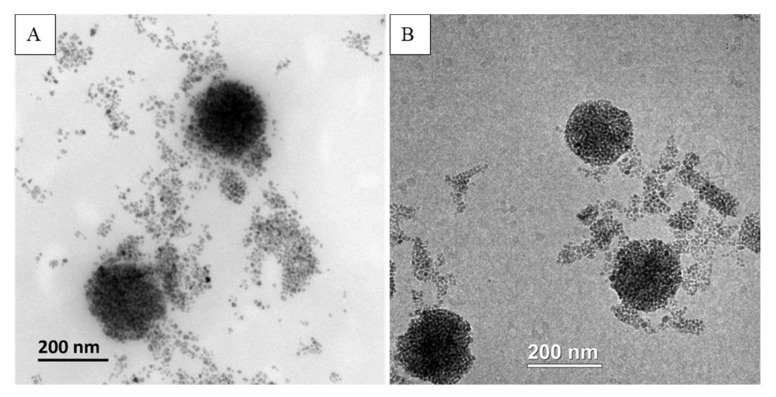
(**A**) TEM and (**B**) crytoTEM of REV_MCLs.

**Figure 12:**
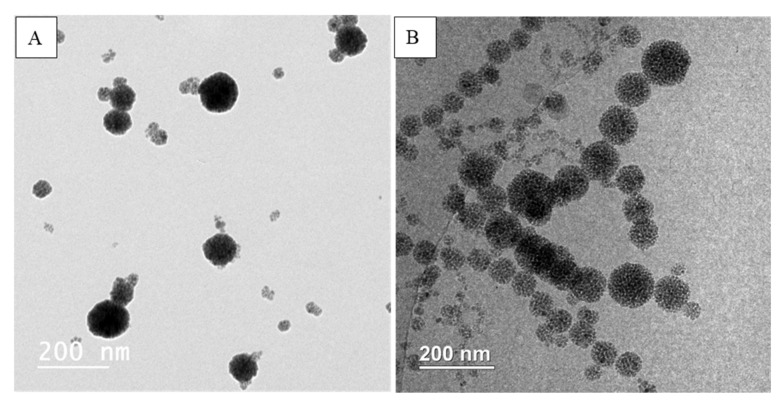
(**A**) TEM and (**B**) CryoTEM of cosol_MCLs.

The two best formulations prepared by 2 different methods were compared. From Figure 11 and Figure 12, we can observe that both formulations provided MCLs with a high content of MNPs inside liposomes. However, the liposomes obtained with the cosolvent sonication method tend to be more homogenous, and less free MNPs would be observed as compared to the one obtained by reverse phase evaporation. These results are consisted with the lower PDI value obtained for cosol_MCLs as compared to REV_MCLs (Table 2 and Table 3).

Nanoparticles were well known for their ability to enhance the accumulation of drug delivery system in tumor through the enhanced permeability and retention (EPR) effect. The EPR effect is driven by the difference between the microenvironment in the tumor and normal tissues, including the enhanced permeability of tumor vessels, and the dysfunction of the tumor lymphatic drainage leading to a higher retention time of the drug delivery system in the tumor tissue. The hyper-permeability of the tumor is due to the leakage of about hundreds of nanometers between endothelial cells [29]. Meanwhile, junctions between endothelial cells in normal vessels are tight, with the gap of less than 10 nm [30]. These differences allow nanoparticles of up to about 400 nm in diameter to passively target tumor tissue [31]. Therefore, our MCLs with a diameter of about 200 nm would be suitable for passive targeting purposes through the EPR effect.

#### 3.4.2. Movement of MCLs under External Magnetic Field Exposure

**Figure 13:**
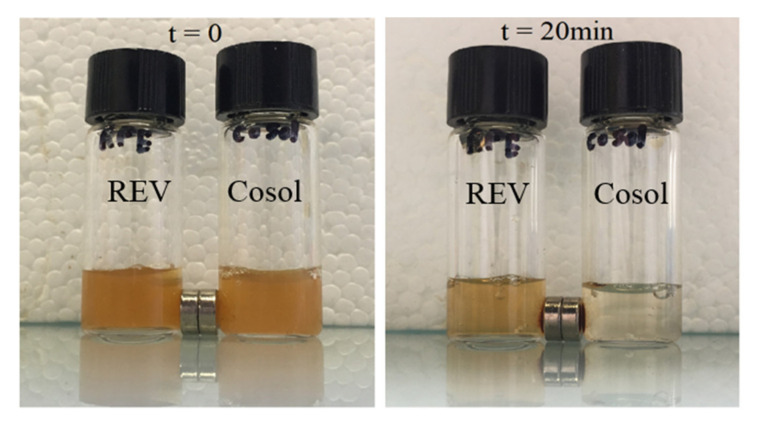
Attraction of MCL formulations when exposed to a magnet. REV_MCLs and cosol_MCLs were put in 2 vials at a concentration of 5 mM of Fe. Two small NdFeB magnets (0.34 T) were placed next to the vials for 20 min. REV:MCLs by reverse phase evaporation method; cosol: MCLs by the cosolvent sonication method.

In order to show the possibility of magnetic targeting, the accumulation of MCLs under exposure to a magnetic field gradient was carried out using two small NdFeB magnets placed close to the vial containing MCLs prepared by the reverse phase evaporation method or by the cosolvent sonication method. As can be seen on Figure 13, with the same concentration of iron of 5 mM, cosol_MCLs moved to the side of the magnet much faster than REV_MCLs. This means that the cationic magnetic liposomes prepared by cosol are more magnetic (more MNPs per liposome) than the REV ones.

#### 3.4.3. MRI Relaxivity at 7T

**Figure 14:**
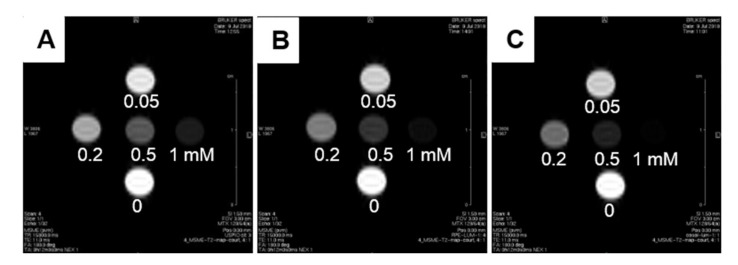
*T2* weighted MRI at 7 T of: (**A**) free MNPs; (**B**) REV_MCLs; and (**C**) cosol_MCLs at various iron concentrations in water.

The relaxivities of the two formulations of REV_MCLs and Cosol_MCLs were measured at 7 T and compared to free MNPs (Figure 14). Iron oxide NPs are a *T2* contrast agent that give a hyposignal in *T2* weighted MRI. The higher the *r_2_* is, the better contrast enhancement of the agent. In our work, both formulations enhanced *r_2_* and *r_2_/r_1_* compared to the free MNPs. It was reported that a greater level of MNPs encapsulated into a liposomes induced stronger *T2* relaxation time shortening compared to free particles [32]. The much higher *r_2_* of the cosol_MCLs in comparison with REV_MCLs (Table 5) was a result of the more homogenous liposomes with less free particles, and a higher amount of MNPs per liposome was in coherence with the faster movement of MCLs under external magnetic field exposure. Therefore, cosol formulation could be a good candidate for MRI imaging guided gene delivery.

#### 3.4.4. Lipoplexation

Lipoplex formation was evidenced by Picogreen assay (Table 6). The high complexation efficiency of about 90–95% of the two formulations confirmed that they can form complexes with plasmid DNA at various charge ratios. The lipoplexes at the lowest charge ratio of RC8 were chosen for transfection into a CT26 cell line to minimize the possible toxicity caused by cationic lipid [33,34].

Lipoplexes formation and lipoplexation efficiency calculation were described above. The data given are the averages of 3 different experiments (*n* = 3), bars, SD. Ctrl+: positive control (liposome DOPE/DMAPAP/C14PEG1000 49:50:1 mol/mol); RC = nmol DMAPAP/µg pFAR4-luc; LE: lipoplexation efficiency.

#### 3.4.5. In Vitro Transfection Efficiency

**Figure 15:**
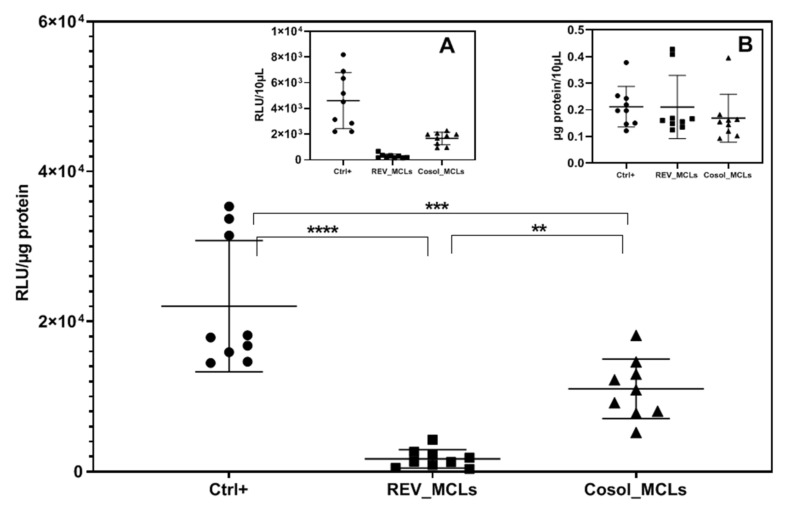
Transfection efficiency of lipoplexes at RC8 in a CT26 cell line. Cells were seeded at 10^4^ cells/well in a 96-well plate. Luciferase and protein assay were carried out 24 h after the incubation of cells with 100 µL of lipoplexes containing 1 µg of pFAR4-luc in the absence of a magnetic field for 3 h. Transfection efficiency is calculated as RLU/µg protein. The data given are averages of 3 different experiments, each performed in triplicate (*n* = 9); bars, SD. One-way ANOVA was done with GraphPad Prism software, ** *p* ≤ 0.01, *** *p* ≤ 0.001, ****p ≤ 0.0001. Inserted figure: (**A**) RLU, (**B**) µg protein in 10 µL of cell lysis 24 h after incubation. RLU, relative light unit; RC = nmol DMAPAP/µg pFAR4-luc. Ctrl+ (dark circle): positive control (lipoplexes based on liposome DOPE/DMAPAP/C14PEG1000 49:50:1 mol/mol); REV_MCLs (dark square): lipoplexes based on REV_MCLs; cosol_MCLs (dark triangle): lipoplexes based on cosols MCLs.

The transfection efficiency of lipoplexes were calculated as luminescence unit per µg of protein (RLU/µg protein). We found that cosol_MCLs (~2 mg/mL of Fe_2_O_3_) transfected at a level 6 times higher than REV_MCLs (approximately 1.6 mg/mL of Fe_2_O_3_) (Figure 15). Meanwhile, their transfection was 2 times lower than the positive control liposome DOPE/DMPAP/C14PEG1000. The inefficient transfection with REV_MCLs could be due to their much higher cytotoxicity to the CT26 cells (Figure S3) compared to that of control liposomes and cosol_MCLs. This toxicity could come from the remaining CHCl_3_ and diethyl ether, which may be not completely removed. Besides, the lower transfection efficiency of cocol_MCLs than the control liposomes could be due to the loss of cationic lipids during the preparation and purification of cosol_MCLs shown by the lower zeta potential of liposomes and lipoplexes (Table S5).

It is reported in some publications that at a high level of encapsulated MNPs, a low transfection efficiency of MCLs could be obtained. For example, Zheng et al. proved that MCLs with a high MNPs content of 1.5–3 mg/mL had a much lower transfection than that of 0.75 mg/mL and non-magnetic liposomes [16]. Samadikhah also confirmed that the formulation with 1 mg/mL of MNPs had about 4 times lower transfection compared to that of 0.5 mg/mL, which was at the same transfection level as the cationic liposomes [17]. Therefore, we continued working on cosol_MCLs for further studies.

### 3.5. Optimization of In Vitro Transfection by Cosol_MCLs

#### 3.5.1. Optimization of PEG Length and Charge Ratio

**Figure 16:**
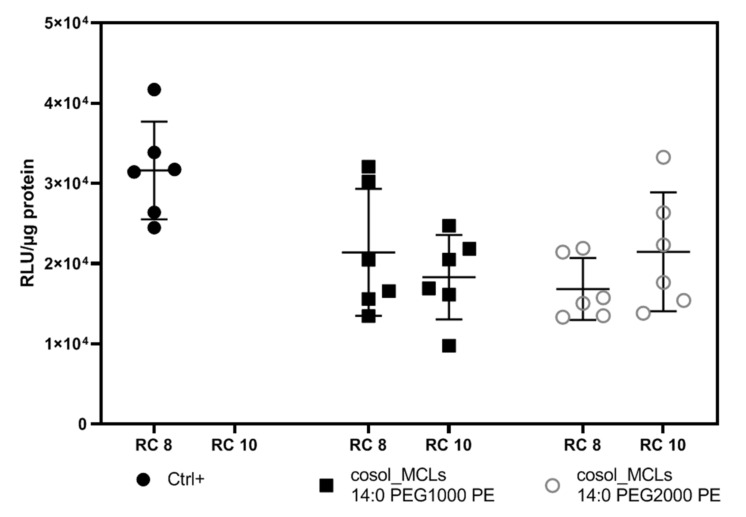
Transfection efficiency of lipoplexes based on cosol_MCLs with different PEG-PE in CT26 cell line. Cells were seeded at 10^4^ cells/well in a 96-well plate. Luciferase and protein assay were carried out 24 h after incubation of cells with 100 µL of lipoplexes containing 1 µg of pFAR4-luc in the absence of magnetic field for 3 h. Transfection efficiency is calculated as RLU/µg protein. The data given are averages of 2 different experiments, each performed in triplicates (*n* = 6); bars, SD. RLU, relative light unit; RC = nmol DMAPAP/µg pFAR4-luc. Ctrl+ (dark circle): positive control (lipoplexes based on liposome DOPE/DMAPAP/C14PEG1000 49:50:1 mol/mol); cosol_MCLs: lipoplexes based on cosols MCLs with 1% of 14:0 PEG1000-PE (dark square) or 14:0PEG2000-PE (clear circle).

It was shown in other works that the charge ratio between cationic liposomes and plasmid DNA strongly affects the size, zeta potential, stability, toxicity, and therefore the transfection efficiency of the lipoplexes [35,36,37]. Figure 16 presents the transfection efficiency of cosol_MCLs–pFAR4-luc complexes with different PEG lengths at a charge ratio of 8 or 10. We can see that an increased ratio from 8 to 10 did not affect the level of protein nor the transfection level. The addition of PEG is known to result in a lower level of transfection, which is probably due to a lower internalization of the lipoplexes [38]. Nevertheless, the increase in PEG length from 1000 to 2000 did not alter the transfection efficiency of cosol_MCLs.

#### 3.5.2. Optimization of Magnetic Induction Time

Based on all of the results from characterization to in vitro transfection, we chose the MCLs prepared by the cosolvent sonication method with 1% of 14:0 PEG1000-PE for further studies. Next, the transfection efficiency of lipoplexes based on cosol_MCLs was carried out with a support of an external magnetic field for various incubation times.

**Figure 17:**
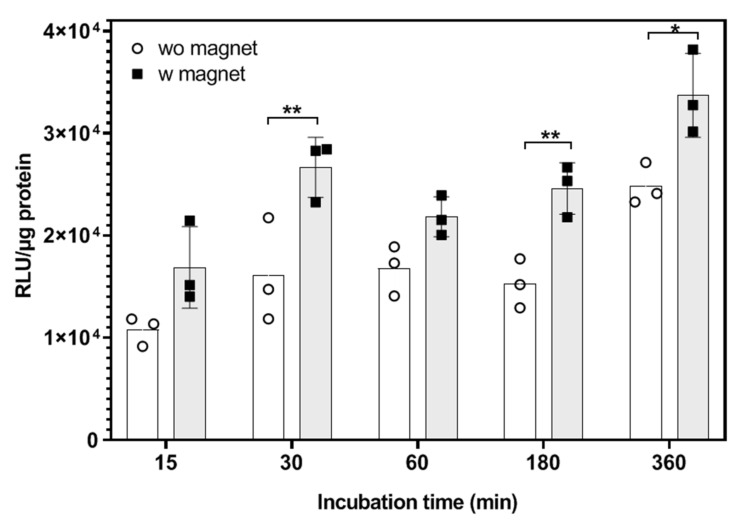
Effect of magnetic field exposure on the transfection efficiency of lipoplexes (cosol_MCLs/pFAR4-luc, RC8) in a CT26 cell line for different incubation times. Cells were seeded at 10^4^ cells/well in a 96-well plate. Luciferase and protein assay were carried out 24 h after the incubation of cells with 100 µL of lipoplexes containing 1 µg of pFAR4-luc in the absence (clear circle) or presence (dark circle) of a magnetic field for various incubation times. The transfection efficiency is calculated as RLU/ug protein. The data given are averages of triplicates of one experiment (*n* = 3); bars, SD. Two-way ANOVA was done with GraphPad Prism software, *p ≤ 0.05, ** *p* ≤ 0.01. RLU, relative light unit; RC = nmol DMAPAP/µg pFAR4-luc.

The transfection with cosol_MCLs at RC8 was highly efficient even after exposure to the lipoplexes for only 15 min, as indicated in Figure 17. When increasing the transfection time, the transfection efficiency increased. Moreover, in the presence of a magnetic field, the transfection was enhanced, even though not always significantly. The highest enhancement of magnetofection by 1.6 times was obtained with 30 min or 180 min of magnetic induction.

This can be explained thanks to the magnetic force created by an external magnetic field; lipoplexes were accelerated to accumulate at the bottom of the well, leading to an increased interaction with the adherent cells. However, with the formulation, the big particles formed when the lipoplexes was dispersed in the media containing serum rapidly sedimented on the surface of the cells (Figure S4). We supposed that the magnetic field just accelerated the accumulation of the particles on the cell surface, but it did not promote the cellular uptake. Therefore, the role of the magnetic field in this case was not highlighted.

Improved transfection with magnetic induction was evaluated in other publications. Using MCLs prepared by the reverse phase evaporation method at an MNP concentration of 0.75/1.5/3 mg/mL, Zheng et al. figured out the increase of transfection of about 2–2.8 times with magnetofection compared to that without a magnetic field. This enhancement, which is slightly higher than our results, may came from the difference in size and zeta potential of the lipoplexes. Indeed, our lipoplexes had the size of about 300 nm with a zeta potential of about 24 mV (Table S5), which was bigger and less positive than the lipoplexes in the work of Zheng (about 200–238 nm in size and 35–38 mV in zeta potential). Moreover, the ratio of lipid and MNPs is different. Their formulation had a lower content of MNPs compared to ours (LP/MNP of 5 and 1.7 w/w respectively) [16]. The effect of MCLs size on transfection could be also found in the work of Jiang et al., in which they indicated that MNPs with size of 200–300 nm had lower transfection efficiency (about 1.3–1.5 times) than that of 50–100 nm MNPs [15]. Another factor that contributes to magnetic induction enhanced-transfection is the dose of transfection vector [39]. Jiang et al. showed that the highest increase in transfection efficiency using magnetic induction was obtained with the lowest plasmid DNA dose of only 25 ng, and they found that it was 2 times less efficient with 100 ng of plasmid DNA [15]. All of these reasons can explain why the similar system prepared by Nakimi et el. provided a much higher enhancement of magnetic induction transfection compared to our formulation. In their work, the system with oleic acid-coated magnetite nanocrystal cores and cationic lipid shells with a very small size of about 40 nm was much more efficient when transfected with a magnet compared to that of the non-magnetic transfection. Beside the particle size, the DNA dose (50 ng) and Fe_3_O_4_ content (about 1 µg) for 10^4^ cells of their treatment were much lower than that of this work (1 µg of plasmid DNA and 16 µg of Fe_2_O_3_ for 10^4^ cells) [40].

Nevertheless, these cationic magnetic lipoplexes have been conceived for in vivo transfection. Their effect had to be shown in vitro, despite the fact that discrepancies have been often observed between in vitro and in vivo results for lipoplexes transfection. The obtention of long-circulating liposomes require PEGylation, while PEG reduces the internalization of the lipoplexes and their transfection efficiency. The conception of magnetic liposomes as an MRI contrast agent requires a sufficient amount of iron oxide nanoparticles, which is deleterious for in vitro transfection efficiency. Finally, no effect of in vivo transfection could be obtained with a low amount of DNA, which usually requires µg of plasmid. Here, we ought to use a small plasmid that already showed its ability to transfect the liver after a single injection of positively charged microbubbles in vivo [41].

### 3.6. Cytotoxicity of Cosol_MCLs and their Lipoplexes

**Figure 18:**
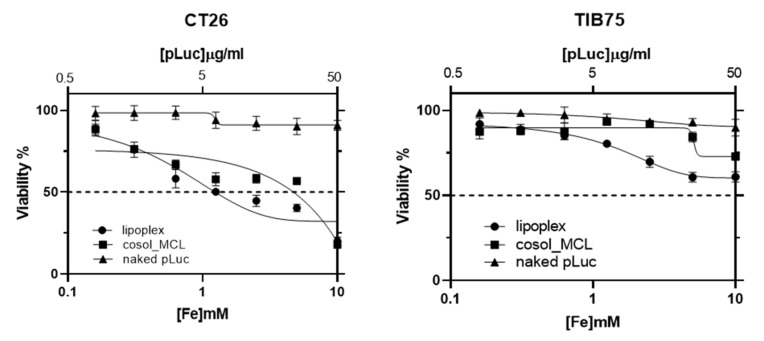
Viability of CT26 (colon cancer) and TIB-75 (hepatocyte) cells after 24 h incubation with naked plasmid DNA (pFAR4-luc), cosol_MCLs, or lipoplexes (RC8). CT26 or TIB75 cells were seeded at 2 × 10^4^ cells/well in a 96 well plate. Alamar blue test was carried out 24 h after incubation of cells with 100 µL of lipoplexes based on cosol_MCLs or cosol_MCLs or naked pFAR4-luc containing various concentration of pFAR4-luc and MNPs. The data given are the averages of 2 different experiments performed in triplicates (*n* = 6); bars, SD.

Cationic liposomes were reported to be toxic for cells due to their interaction with negatively charged cellular components (opsonin, serum protein, and enzyme) resulting in hemolysis, an impairment of mitochondrial function, and membrane integrity in vitro [42]. Our goal was using MCLs–plasmid DNA complexes for in vivo application on CT26 tumor-bearing mice—a well-studied mouse model in our lab for anticancer therapy. Moreover, with the size of more than 200 nm, the lipoplexes were predicted to be captured mostly by the liver. Therefore, the cytotoxicity of our MCLs on colon cancer (CT26) cells and hepatic (TIB75) cells was carried out with Alamar blue test. In both cell lines, naked pLuc seemed to be not toxic with a cell viability of nearly 100%, even at high concentration. On the other hand, cosol_MCLs and their lipoplexes were toxic to CT26 cells, especially at high concentration. In the case of TIB75 cells, they were obviously less toxic (Figure 18).

## 4. Conclusions

MCLs have been studied for a long time for hyperthermia, drug delivery [43], or gene delivery purposes. With positive charge, CLs were known for their ability to bind and interact with negatively charged proteins on a cell membrane, leading to cellular uptake and release of the encapsulated gene [16]. Moreover, MNPs incorporated in MCLs were shown to enhance in vitro transfection under exposure to a magnetic field [15,16,17]. They can also act as a targeting agent using an external magnet [16,43] or heating agent for hyperthermia [44]. However, MCLs were not shown as a contrast enhancement agent for MRI even in vitro or in vivo. In this study, MCLs with a homogeneous size of less than 200 nm were prepared by 2 different methods, cosolvent sonication or reverse phase evaporation. Both of the formulations gave high r_2_ MRI relaxivity at 7 T and good lipoplexation with pFAR4-luc. We found that cosol_MCLs were superior to REV_MCLs in both physicochemical properties and in vitro transfection efficiency. Here, the effect of magnetic induction for enhanced transfection was also studied for different magnetic field exposure times. The highest transfection enhancement of about 1.6 times was obtained with a magnetic induction time of 30 min or 3 h.

In conclusion, we have presented a work on MCLs with effective in vitro transfection efficiency and MRI contrast enhancement. In addition, our MCLs-plasmid DNA lipoplexes seemed to be poorly toxic to hepatic cell line. More work should be done to optimize their in vivo stability, magnetic targeting, and transfection.

## Figures and Tables

**Table 1:** Lipid components and iron concentration for magnetic liposome formulations. DOPE: 1,2-dioleoyl-sn-glycero-3-phosphoethanolamine, DPPC: 1,2-dipalmitoyl-sn-glycero-3- phosphocholine, DSPC: 1,2-distearoyl-sn-glycero-3-phosphocholine, PEG-PE: 1,2-dimyristoyl -sn-glycero-3-phosphoethanolamine-*N*-[methoxy(polyethylene glycol)].

Components	MCLs	UMLs
DOPE (mol%)	49	0
DMAPAP (mol%)	50	0
14:0 PEG-PE (mol%)	1	0
DPPC (mol%)	0	86
DSPC (mol%)	0	9
18:0 PEG2000-PE (mol%)	0	5
Fe (mM)	2.5–1000	1000

**Table 2:** Characterizations of magnetic cationic liposomes (MCLs) prepared by reverse phase evaporation method using various amounts of magnetic nanoparticles (MNPs) (γ-Fe_2_O_3_).

Label	Fe Concentration Input (mM)	Z Average (d.nm)	PDI	Zeta Potential (mV)	Encapsulation Efficiency %	Loading Efficiency %
REV_MCL_2.5	2.5	126.3 ± 2.6	0.188 ± 0.043	+71.6 ± 4.2	70.3 ± 2.1	8.3 ± 0.2
REV_MCL_5	5	169.9 ± 24.7	0.202 ± 0.012	+69.1 ± 2.3	75.6 ± 2.3	16.3 ± 0.4
REV_MCL_10	10	194.1 ± 14.2	0.240 ± 0.024	+68.3 ± 4.0	78.8 ± 7.2	28.9 ± 1.9
REV_MCL_25	25	371.8 ± 36.7	0.388 ± 0.037	+57.5 ± 5.4	35.6 ± 7.2	31.3 ± 4.4

Abbreviation: Polydispersity Index, PDI.

**Table 3:** Characterizations of MCLs by the cosolvent sonication method for different sonication times.

Label	Sonication Time (h)	Z Average (d.nm)	PDI	Zeta Potential (mV)	Encapsulation Efficiency %	Loading Efficiency %
Cosol_MCL_1 h	1	268.0 ± 7.2	0.168 ± 0.029	+37.7 ± 0.7	61.0 ± 2.5	37.5 ± 1.0
Cosol_MCL_3 h	3	215.7 ± 15.3	0.158 ± 0.047	+46.8 ± 7.3	67.0 ± 2.7	39.7 ± 1.0
Cosol_MCL_6 h	6	208.8 ± 24.8	0.178 ± 0.052	+47.1 ± 2.5	69.4 ± 3.7	40.6 ± 1.3

**Table 4:** Characterizations of MCLs by the cosolvent sonication method using different PEG-PE.

PEG-PE	Z Average (d.nm)	PDI	Zeta Potential (mV)
14:0 PEG750 PE	175.2 ± 20.2	0.143 ± 0.004	49.5 ± 1.4
14:0 PEG1000 PE	207.4 ± 11.7	0.143 ± 0.022	41.7 ± 0.1
14:0 PEG2000 PE	244.4 ± 66.3	0.184 ± 0.033	44.5 ± 0.8

**Table 5:** Calculated relaxivities of free MNPs, REV_MCLs, and cosol_MCLs at various concentrations of iron in water at 7 T.

Sample	*r_1_* (s^−1^.mM^−1^)	*r_2_* (s^−1^.mM^−1^)	*r_2_/r_1_*	r_2_* (s^−1^.mM^−1^)
Free MNPs	2.54	159.13	62.58	178.87
REV_MCLs	0.37	172.77	461.83	279.18
Cosol_MCL	1.07	222.59	207.99	296.74

*r*_1_: longitudinal relaxivity; *r*_2_: transverse relaxivity; *r*_2_*: effective transverse relaxivity.

**Table 6:** Lipoplexation efficiency calculated from Picogreen assay.

Sample	RC	LE %
Ctrl+	8	92.0 ± 3.8
REV_MCLs	8	90.5 ± 5.1
	10	94.9 ± 1.8
	12	93.9 ± 1.6
Cosol_MCLs	8	88.0 ± 3.7
	10	87.8 ± 5.1
	12	89.0 ± 2.4

## Supplementary Materials

The following are available online at https://www.mdpi.com/1999-4923/12/9/854/s1, Figure S1: TEM images of UMLs after insertion of 40% of DMAPAP at various pH, Figure S2: TEM images of UML samples after insertion of 40% of DMAPAP: (A) in water or (B) in CHCl_3_, Figure S3: Viability of CT26 cells after transfection with various lipoplexes at RC8, Figure S4: Stability of cosol_MCLs/pLuc lipoplexes at RC8 in culture medium, Table S1: Hydrodynamic size and zeta potential of γ-Fe_2_O_3_ MNPs in water, Table S2: Characterization of UMLs after addition of different amount of DMAPAP, Table S3: Characterization of post insertion—UMLs at different pH, Table S4: Characterization of post insertion—UMLs at 2 different temperatures and solvents, Table S5: Size, PDI and zeta potential of various liposomes and their lipoplexes at RC8 in H_2_O.
