# Supplementary material for: Development of Theranostic Cationic Liposomes Designed for Image-Guided Delivery of Nucleic Acid
Source: Pharmaceutics. 2020 Sep 8;12(9):854. doi: 10.3390/pharmaceutics12090854 (PMC7559777; doi:10.3390/pharmaceutics12090854)
Supplement: Supplementary file 1 [file pharmaceutics-12-00854-s001.pdf]

pharmaceutics

Supplementary Materials: Development of Theranostic Cationic Liposomes Designed for Image-Guided Delivery of Nucleic Acid

Hai Doan Do, Christine Ménager, Aude Michel, Johanne Seguin, Tawba Korichi, Hélène Dhotel, Corinne Marie, Bich-Thuy Doan and Nathalie Mignet

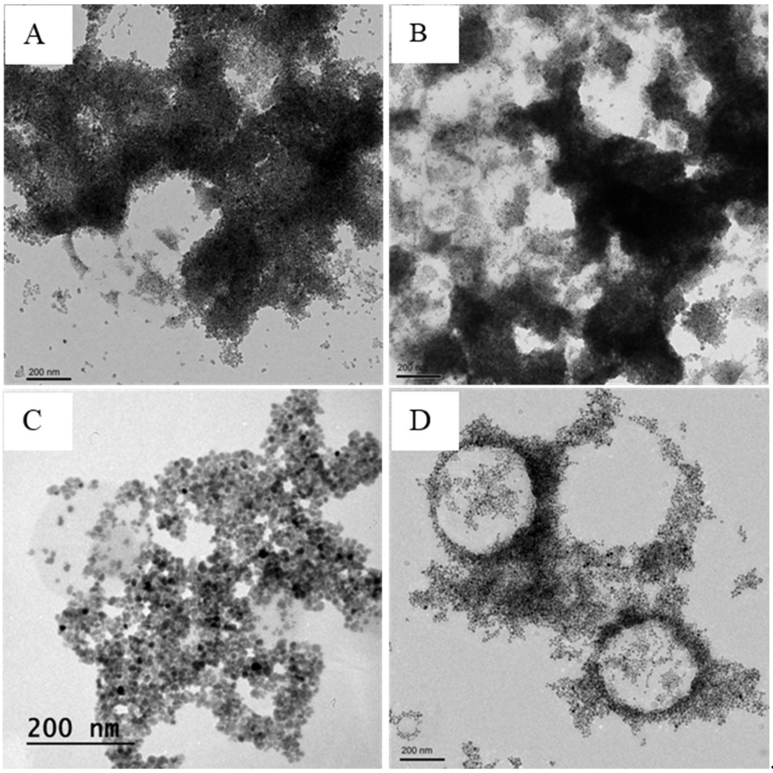

Figure S1. TEM images of UMLs after insertion of 40% of DMAPAP at various pH: (A) pH 5, (B) pH 7, (C) pH 9 and (D) pH 11. 40 % of DMAPAP per total lipid was added to a diluted dispersion of UMLs (50mM of Fe, 0.36 μmol total lipids) at various pH. After stirring for 1h, centrifugation and magnetic separation, TEM images of the post-inserted UMLs was observed.

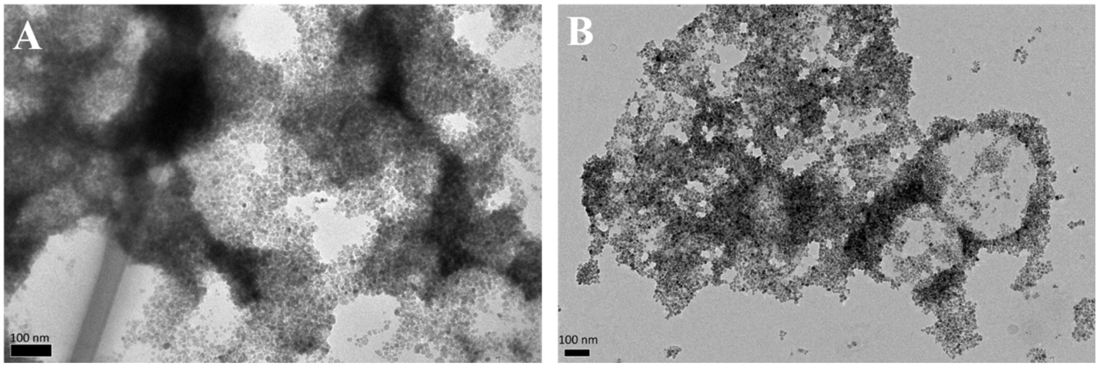

Figure S2. TEM images of UML samples after insertion of 40% of DMAPAP: (A) in water or (B) in $CHCl_3$. 40 % of DMAPAP per total lipid was added to a diluted dispersion of UMLs (50mM of Fe, 0.36

µmol total lipids). After stirring for 1h, centrifugation and magnetic separation, TEM images of the post-inserted UMLs was observed.

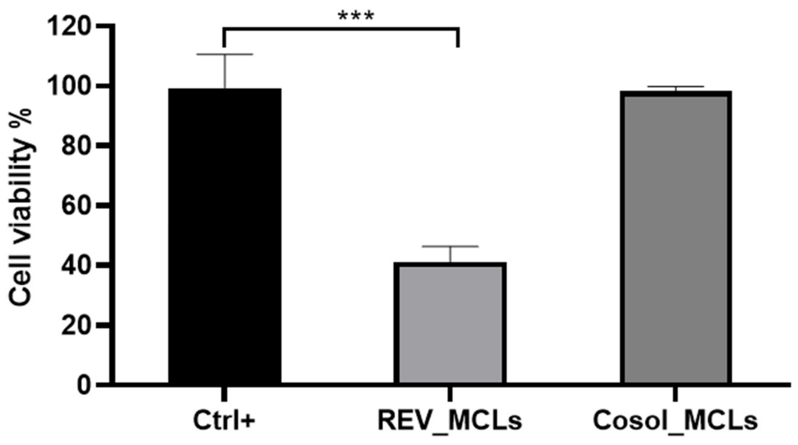

Figure S3. Viability of CT26 cells after transfection with various lipoplexes at RC8.

CT26 cells were seeded at 10^4 cells/well in a 96 well plate. Alamar blue test was were carried out 24h after incubation of cells with 100 µL of lipoplexes containing 1 µg of pFAR4-luc in the absence of magnetic field for 3h. The data given are averages of 3 different experiments (n=3); bars, SD. One way ANOVA was done with GraphPad Prism software, *** P≤0.001. Ctrl+: positive control (lipoplexes based on liposome DOPE: DMAPAP: C14PEG1000 49:50:1 mol/mol); REV_MCLs: lipoplexes based on REV_MCLs; cosol_MCLs: lipoplexes based on cosols MCLs.

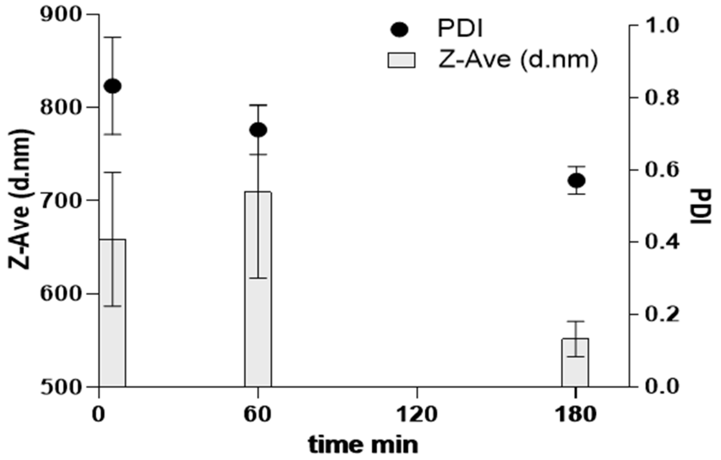

Figure S4. Stability of cosol_MCLs/pFAR4-luc lipoplexes at RC8 in culture medium. Cosol_MCLs/pFAR4-luc lipoplexes at RC8 were prepared as described above. Then the lipoplexes were diluted 5 times in complete DMEM containing 10% FBS and incubated at 37°C. Hydrodynamic size was measured by DLS after 0h, 1h or 3h incubation. The data given are the averages of 3 measurement of one experiment (n=3); bars, SD.

Table S1. Hydrodynamic size and zeta potential of γ-Fe_2O_3 MNPs in water.

Size d.nm	PDI	Zeta Potential
22.6 ± 0.3	0.194 ± 0.039	−26.2 ± 4.7

Hydrodynamic size and zeta potential were measured 3 times/ sample, the mean and SD of three means of the 3 measurements were given; bars, SD.

Table S2. Characterization of UMLs after addition of different amount of DMAPAP.

Label	Preformed UMLs	PI_UMLs_pH_10	PI_UMLs_pH7_20	PI_UMLs_pH7_40
% of DMAPAP	-	10	20	40
Liposome peak nm (intensity %)	175.8 (22.3%)	106.1 (28.4%)	152.9 (42%)	134.1 (57.1%)
Zeta potential (mV)	-45.4	-38.4	-33.8	-36.3
Aggregate	-	-	+	++
Stability	Stable	Stable	Stable	Less stable

Table S3. Characterization of post insertion – UMLs at different pH.

Label	PI_MCL pH3	PI_MCL pH5	PI_MCL pH7	PI_MCL pH9	PI_MCL pH11
% of DMAPAP	40	40	40	40	40
pH	3	5	7	9	11
Liposome peak (nm) (intensity %)	204.3 (100%)	139.0 (33.3%)	134.1 (57.1%)	81.5 (42%)	105.9 (71.2%)
Zeta potential (mV)	-28.2	-25.3	-36.3	-25.8	-20.8
Aggregate	++	-	+	-	-

Table S4. Characterization of post insertion - UMLs at 2 different temperatures and solvents.

DMAPAP Solution	DMAPAP in H_2O		DMAPAP in $CHCl_3$	
T°	45°C	RT	45°C	RT
Liposome peak nm (intensity %)	185.2 (42.3%)	167.6 (70.2%)	163.0 (42.8%)	197.2 (34.5%)
Zeta potential (mV)	-20.3	-19.8	-22.2	-22.4
Aggregate	+	+	+	+
Stability	Stable	Stable	Stable	Stable

RT: Room temperature.

Table S5. Size, PDI and zeta potential of various liposomes and their lipoplexes at RC8 in H_2O.

Cationic Liposome	Control		REV_MCLs		Cosol_MCLs	
pFAR4-luc	-	+	-	+	-	+
Size d.nm	88.21 ± 6.5	130.0 ± 10.5	206.4 ± 1.4	202.9 ± 2.0	188.1 ± 5.6	301.3 ± 26.7
PDI	0.295 ± 0.025	0.342 ± 0.038	0.249 ± 0.016	0.293 ± 0.029	0.127 ± 0.017	0.342 ± 0.038
Zeta potential mV	+66.4 ± 1.7	+53.4 ± 4.1	+64.8 ± 1.5	+57.3 ± 5.7	+45.7 ± 6.7	+24.0 ± 5.3

Control: liposome DOPE: DMAPAP: C14PEG1000 (49:50:1 mol/mol). Hydrodynamic size and zeta potential were measured 3 times/ sample, the mean and SD of three means of the 3 measurements were given; bars, SD.

© 2020 by the author. Licensee MDPI, Basel, Switzerland. This article is an open access article distributed under the terms and conditions of the Creative Commons Attribution (CC BY) license (http://creativecommons.org/licenses/by/4.0/).
